# Interleukin-1β causes excitotoxic neurodegeneration and multiple sclerosis disease progression by activating the apoptotic protein p53

**DOI:** 10.1186/1750-1326-9-56

**Published:** 2014-12-12

**Authors:** Silvia Rossi, Caterina Motta, Valeria Studer, Giulia Macchiarulo, Elisabetta Volpe, Francesca Barbieri, Gabriella Ruocco, Fabio Buttari, Annamaria Finardi, Raffaele Mancino, Sagit Weiss, Luca Battistini, Gianvito Martino, Roberto Furlan, Jelena Drulovic, Diego Centonze

**Affiliations:** Clinica Neurologica, Dipartimento di Medicina dei Sistemi, Università Tor Vergata, Via Montpellier 1, 00133 Rome, Italy; IRCCS Istituto Neurologico Mediterraneo (INM) Neuromed, Pozzilli, Italy; Fondazione Santa Lucia/Centro Europeo per la Ricerca sul Cervello (CERC), Rome, Italy; Neuroimmunology Unit, Institute of Experimental Neurology (INSpe), Division of Neuroscience, San Raffaele Scientific Institute, Milan, Italy; Clinica Oculistica, Dipartimento di Biopatologia, Università Tor Vergata, Rome, Italy; Clinic of Neurology, Faculty of Medicine, University of Belgrade, Belgrade, Serbia

**Keywords:** Apoptosis, Glutamate, Inflammation, MS, Synaptic transmission, Tumor necrosis factor-α

## Abstract

**Background:**

Understanding how inflammation causes neuronal damage is of paramount importance in multiple sclerosis (MS) and in other neurodegenerative diseases. Here we addressed the role of the apoptotic cascade in the synaptic abnormalities and neuronal loss caused by the proinflammatory cytokines interleukin-1β (IL-1β) and tumor necrosis factor (TNF-α) in brain tissues, and disease progression caused by inflammation in relapsing-remitting MS (RRMS) patients.

**Results:**

The effect of IL-1β, but not of TNF-α, on glutamate-mediated excitatory postsynaptic currents was blocked by pifithrin-α (PFT), inhibitor of p53. The protein kinase C (PKC)/transient receptor potential vanilloid 1 (TRPV1) pathway was involved in IL-1β-p53 interaction at glutamatergic synapses, as pharmacological modulation of this inflammation-relevant molecular pathway affected PFT effects on the synaptic action of IL-1β. IL-1β-induced neuronal swelling was also blocked by PFT, and IL-1β increased the expression of p21, a canonical downstream target of activated p53.

Consistent with these *in vitro* results, the Pro/Pro genotype of p53, associated with low efficiency of transcription of p53-regulated genes, abrogated the association between IL-1β cerebrospinal fluid (CSF) levels and disability progression in RRMS patients. The interaction between p53 and CSF IL-1β was also evaluated at the optical coherence tomography (OCT), showing that IL-1β-driven neurodegenerative damage, causing alterations of macular volume and of retinal nerve fibre layer thickness, was modulated by the p53 genotype.

**Conclusions:**

Inflammatory synaptopathy and neurodegeneration caused by IL-1β in RRMS patients involve the apoptotic cascade. Targeting IL-1β-p53 interaction might result in significant neuroprotection in MS.

## Background

The immune and central nervous systems are more intimately related than previously supposed. Effector immune cells are regulated by neurotransmitters [1–3], and cytokine signaling in the brain is known to modulate important functions, including neurotransmitter metabolism, neuroendocrine function, synaptic plasticity, as well as the neural circuitry of cognition and mood [4–10]. On the other hand, this link may be the condition for pathological processes. Neuroinflammation is a hallmark of most neurological diseases, such as multiple sclerosis (MS) and other neurodegenerative diseases [11, 12].

Proinflammatory cytokines, such as tumor necrosis factor (TNF-α) and interleukin-1β (IL-1β), have been shown to increase synaptic transmission and to induce excitotoxic neuronal damage [4, 7, 13–15]. Furthermore, we have recently demonstrated that IL-1β plays a role in MS-associated neurodegenerative damage and clinical progression [16]. However the molecular mechanisms of inflammation-driven neurodegeneration are still largely unclear.

Aim of our work was to investigate the possible role of the apoptotic cascade in the inflammatory neurodegeneration *in vitro* and in MS patients. Proinflammatory cytokines are able to induce p53, and are involved in the enhancement of p53-mediated apoptosis [17–22]. In neurons, the tumor-suppressor protein p53 is believed to play roles in physiological apoptosis, as well as in the neuronal death that occurs in disorders such as Parkinson’s disease, Alzheimer’s disease, and stroke [23–27]. Notably, the gene encoding p53 (TP53 gene) presents a common single nucleotide polymorphism (SNP; G-to-C transversion) at codon 72 (rs1042522). The two resulting variants (Arg and Pro) are not functionally equivalent, either biochemically or biologically, with the p53Arg variant being more efficient than the p53Pro to induce apoptosis [28–31].

Investigating how p53 genetic variants influence the synaptic and toxic effects of proinflammatory cytokines might provide further crucial insights into the pathophysiology of the neurodegenerative damage of MS and, possibly, of other neurological diseases.

## Results

### p53 regulates the effects of IL-1β at glutamatergic synapses

**Figure 1:**
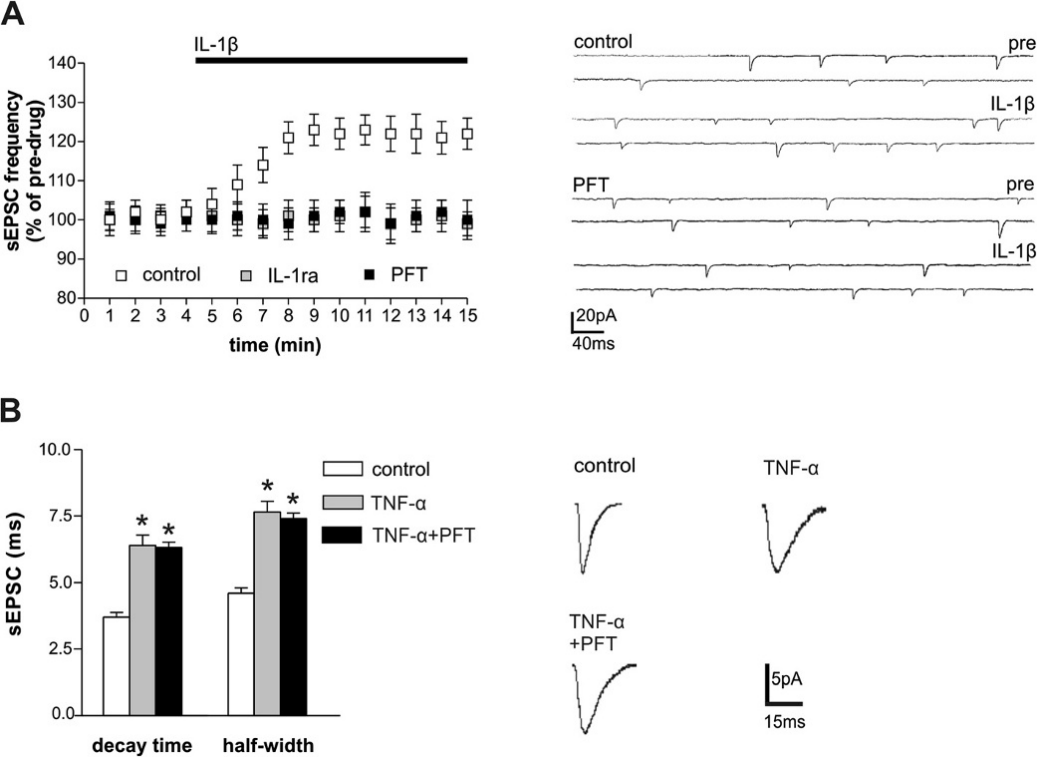
**Role of p53-IL-1β interaction on glutamate synaptic transmission. A**. The graph shows that application of IL-1β, in mice brain slices, significantly enhanced sEPSC frequency (p < 0.05 respect to pre-drug values), an effect fully prevented by both IL-1ra and the p53 inhibitor PFT (p < 0.05 respect to pre-drug values). The electrophysiological traces on the right are examples of sEPSCs recorded from single striatal neurons, before and during the application of IL-1β, in control conditions and in slices pre-treated with PFT. **B**. PFT failed to affect TNF-α effects on sEPSC duration (p > 0.05). The electrophysiological traces on the right are examples of sEPSC mean peak, obtained by group analysis and recorded from striatal neurons in the presence of TNF-α and PFT. *means p < 0.05.

Both IL-1β and TNF-α modulate glutamate-mediated transmission at central synapses [4, 7]. Here, the role of p53 in IL-1β- and TNF-α-mediated synaptic effects were investigated. As reported [7], IL-1β enhanced the frequency of glutamate-mediated spontaneous excitatory post-synaptic currents (sEPSCs) in mouse corticostriatal brain slices (n = 11, p < 0.05 respect to pre-drug values), an effect that was prevented by IL1ra (n = 8, p > 0.05 respect to pre-drug values). We then explored, for the first time, the synaptic effects of IL-1β-p53 interaction. We found that IL-1β failed to enhance sEPSCs in slices incubated with the p53 inhibitor PFT (n = 17, p > 0.05 respect to pre-drug values), indicating the crucial role of p53 in the IL-1β synaptic effects (Figure 1A).

Conversely, the effect of TNF-α on glutamatergic transmission was not mediated by p53. TNF-α, in line with our previous report [4], caused the expected enhancement of sEPSC decay time and half-width in corticostriatal brain slices (n = 11, p < 0.05 respect to control), and here we showed that this effect was still present in PFT-treated slices (n = 17, p < 0.05 respect to control, p > 0.05 respect to TNF-α alone) (Figure 1B). The effect of p53 modulation on TNF-α synaptic effect was never assessed before.

### Involvement of PKC/TRPV1 pathway in IL-1β-p53 interaction at glutamatergic synapses

**Figure 2:**
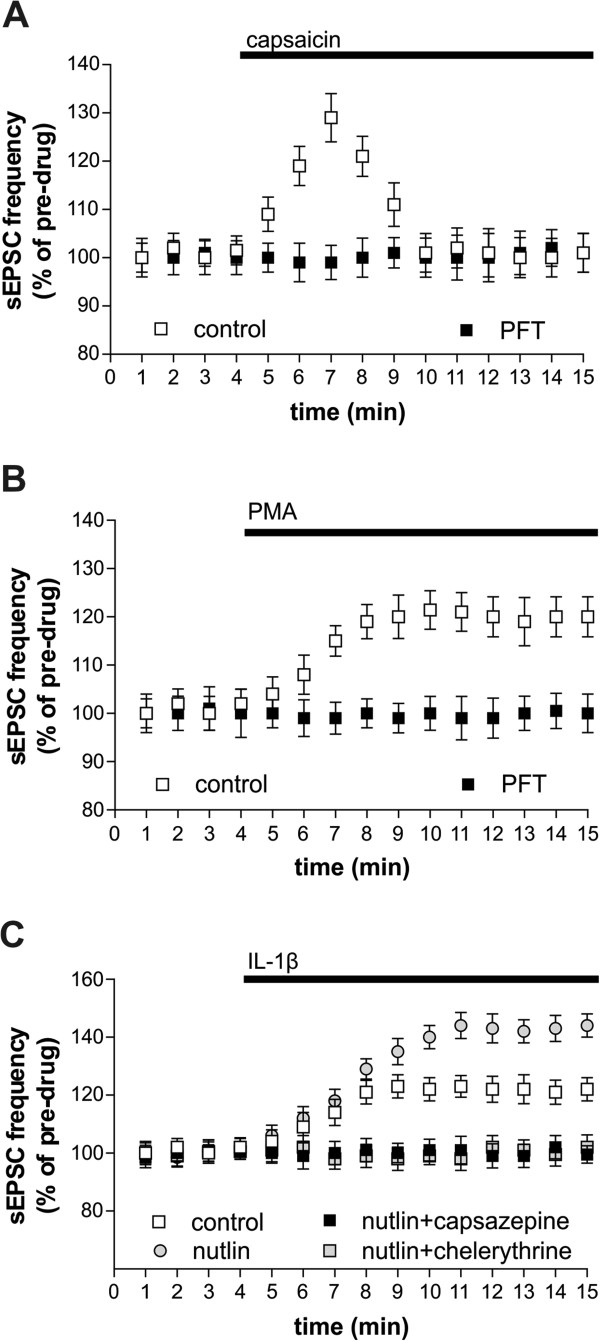
**Role of PKC/TRPV1 pathway in IL-1β-p53 interaction on glutamate synaptic transmission. A**. Capsaicin, agonist of TRPV1 channels, caused a rapid and transient increase of sEPSC frequency in control conditions (p < 0.05) but not in slices pre-treated with PFT (p > 0.05). **B**. PKC activation with PMA was able to mimic IL-1β effects on sEPSC frequency in control slices, but not in presence of PFT. **C**. Nutlin, a p53 activator, enhances the IL-1β effects on sEPSC frequency (p < 0.05 respect to pre-drug value), but not in the presence of capsazepine (p > 0.05), TRPV1 channels antagonist, or chelerythrine (p > 0.05), PKC antagonist.

IL-1β effects on sEPSC are lost after genetic or pharmacological inhibition of TRPV1 channels [7]. The possible role of these channels in the IL-1β-p53 interaction was therefore investigated. Capsaicin, agonist of TRPV1 channels, caused a rapid and transient increase of sEPSC frequency in control conditions (n = 13, p < 0.05), but not in slices pre-treated with PFT (n = 17, p > 0.05), indicating that p53 is fundamental for TRPV1 channel synaptic effects (Figure 2A). IL-1β stimulates PKC at glutamatergic nerve terminals [7], and PKC is a major activator of TRPV1 channels [32]. PKC activation with phorbol 12-myristate 13-acetate (PMA) was able to mimic IL-1β effects on sEPSC frequency in control slices (n = 15, p < 0.05) but not in the presence of PFT (n = 14, p > 0.05), confirming the relevance of PKC/TRPV1 pathway in IL-1β-p53 interaction at glutamatergic synapses (Figure 2B). In line with this, the p53 activator nutlin-3 enhanced the increase of sEPSC frequency mediated by IL-1β (n = 12, p < 0.05), but not in the presence of the TRPV1 antagonist capsazepine (n = 10, p > 0.05) or of the PKC inhibitor chelerythrine (n = 10, p > 0.05) (Figure 2C).

### p53 regulates the in vitro neurotoxic effect of IL-1β

**Figure 3:**
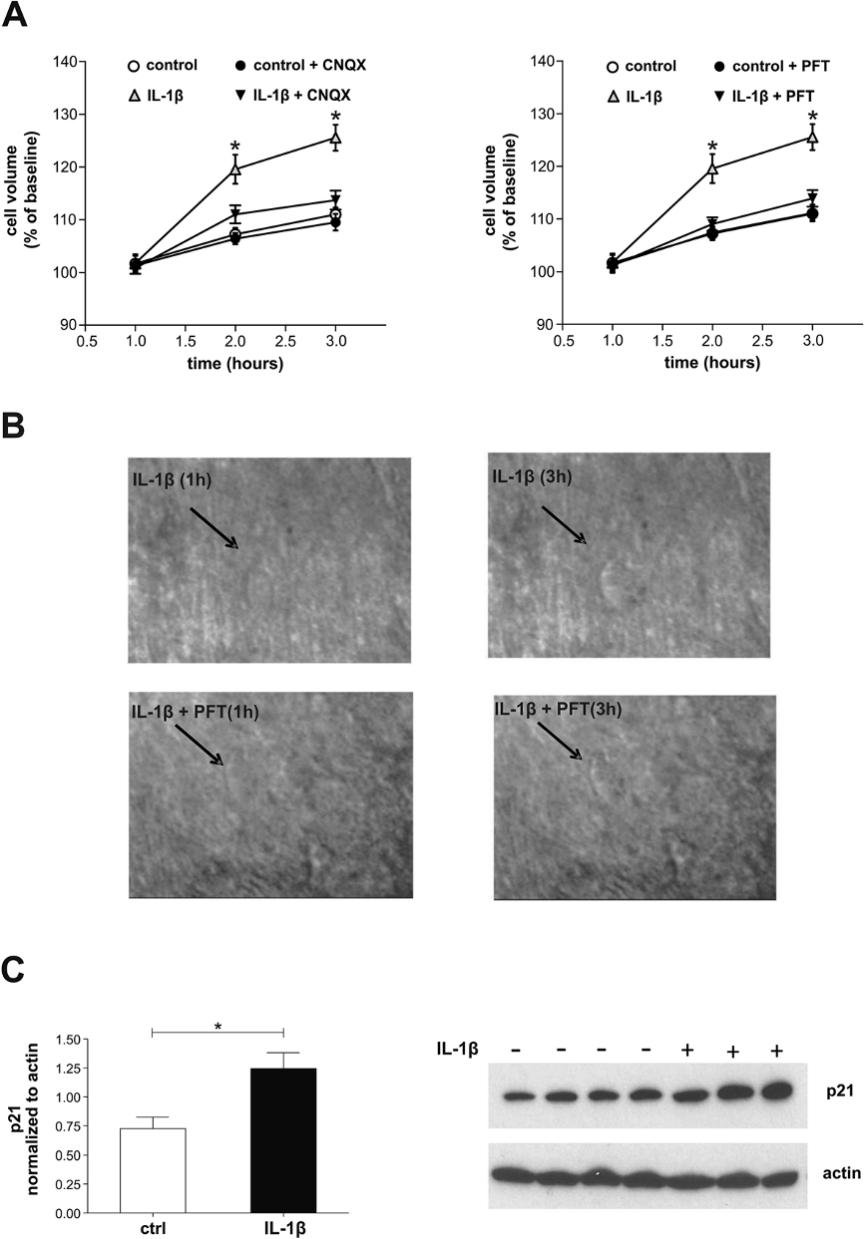
**Role of p53-IL-1β interaction on neuronal damage. A**. The graphs show that time-dependent cell swelling was significantly more pronounced in slices incubated with IL-1β than in control conditions (p < 0.05) and that this effect was attenuated by CNQX (p > 0.05), a glutamate AMPA receptor antagonist, and PFT (p > 0.05). **B**. Examples of neuronal swelling induced by IL-1β in the presence or absence of PFT. Individual neurons were visualized in situ using a differential interference contrast optical system. **C**. p21 protein levels were determined in total protein extracts (300 μg) of mouse corticostriatal slices untreated or treated with IL-1β by Western blot analysis. Each lane represents a different animal and is composed by a triplicate experiment performed on the same animal. Densitometric analysis of each lane was calculated using Image J software and data were normalized to actin used as loading control. IL-1β significantly increases the expression of p21 protein. *means p < 0.05.

Abnormal glutamate transmission causes excitotoxic damage that can be studied in brain slices by measuring neuronal swelling, which reflects the disruption of membrane selective permeability to ions and water [7]. Time-dependent cell swelling was significantly more pronounced in slices incubated with IL-1β (n = 7) than in control conditions (n = 5; p < 0.05). PFT or cyano-nitroquinoxaline-dione (CNQX) attenuated cell swelling in slices treated with IL-1β (n = 10 for both experiments; p < 0.05), confirming that this proinflammatory cytokine causes neuronal damage through an excitotoxic mechanism regulated by p53 (Figure 3A, B).

### IL-1β activates p53 pathway

p53 might play only a permissive role in IL-1β effects or could be activated by IL-1β, thus enhancing synaptic function. In order to evaluate whether IL-1β activates p53 pathway, we analysed the content of p21, which represents a canonical downstream target of activated p53 [33]. We found that IL-1β significantly increases the expression of p21 protein indicating that activation of p53 pathway occurs after IL-1β incubation (Figure 3C).

Of note, the levels of p53 were not detectable in our samples probably due to the continuous proteasome-dependent degradation of p53, as already reported [34].

### Interaction between p53 and IL-1β clinical effects in MS patients

Since p53-dependent apoptosis plays a role in neuronal damage of neurodegenerative diseases [23–27], and can be induced by inflammatory cytokines [17–22], we investigated if a SNP of TP53 gene, known to alter the apoptotic efficiency of p53 [30] could affect the neurotoxic effect of IL-1β in relapsing-remitting MS (RRMS) patients. We have recently demonstrated that the CSF detection of IL-1β in phase of remission predicts disease progression in MS [16]. Comparing between patients with undetectable and detectable CSF levels of IL-1β at baseline, we found that mean Progression Index (PI) and MS Severity Scale (MSSS) were significantly lower among subjects with undetectable IL-1β (PI: 0.19 ± 0.1 versus 0.29 ± 0.2; MSSS: 2.26 ± 2.1 versus 3.41 ± 2.6, p < 0.01 for both parameters).

Then, we grouped patients with detectable levels of IL-1β according to the p53 codon 72 (Arg72Pro) polymorphism, following the dominant and recessive models (Table 1). The analysis of variance showed significant p53 genotype–IL-1β interaction effects on PI and MSSS (Table 2). The Pro/Pro IL-1β + subjects showed lower levels of both PI and MSSS with respect to Arg/Arg and Arg/Pro carriers (PI: 0.09 ± 0.1 versus 0.33 ± 0.19 versus 0.30 ± 0.20; MSSS: 0.91 ± 0.88 versus 4.26 ± 2.58 versus 3.28 ± 2.58), indicating slower disability progression despite exposure to IL-1β. Significant interaction effect for the recessive genetic models was also found, whereas no interaction effect for the dominant model was present (Table 2).

**Table 1:** **TP53 genotype and genetic model frequency distribution in the studied subjects**

TP53 genotype n (%)	IL-1β +	IL-1β -	p
Arg/Arg	39 (50.6)	53 (57.0)	
Arg/Pro	28 (36.4)	29 (31.2)	
Pro/Pro	10 (13.0)	11 (11.8)	0.69
Dominant model n (%)			
Arg/Arg	39 (50.6)	53 (56.9)	
Arg/Pro + Pro/Pro	38 (49.4)	40 (43.1)	0.68
Recessive model n (%)			
Arg/Arg + Arg/Pro	67 (87.0)	82 (88.2)	
Pro/Pro	10 (13.0)	11 (11.8)	0.81

+, detectable; -, undetectable; n, number.

**Table 2:** **p values for p53 gene/IL-1β interaction effects**

	TP53 genotype	dominant model	recessive model
PI	0.002	0.73	0.01
MSSS	0.02	0.30	0.02
MV	0.04	0.16	0.03
RNFL thickness	0.01	0.02	0.005

PI, progression index; MSSS, Multiple Sclerosis Severity Scale; MV, macular volume; RNFL, retinal nerve fiber layer. Although our sample was too small to test other statistical associations, higher values of both PI and MSSS were present in Arg/Arg carriers among patients with undetectable IL-1β (PI: Pro/Pro 0.16 +/- 0.13, Arg/Pro 0.14 +/- 0.09, Arg/Arg 0.21 +/- 0.17; MSSS: Pro/Pro 1.73 +/- 1.62, Arg/Pro 1.73 +/- 1.68, Arg/Arg 2.61 +/- 2.33).

### Interaction between p53 and IL-1β neurotoxic effects in MS patients

The analysis of covariance, adjusted for disease duration, showed significant p53 genotype–IL-1β interaction effects on macular volume (MV) and retinal nerve fibre layer (RNFL) thickness, indicating that also neuronal damage in IL-1β + group was modulated by the Pro/Pro genotype. The analysis of covariance for the recessive models highlighted a significant interaction effect with IL-1β on MV and RNFL thickness (Table 2). Pro/Pro IL-1β + subjects showed higher levels of MV and RNFL thickness with respect to Arg/Arg and Arg/Pro carriers (MV: 7.35 ± 0.87 versus 6.42 ± 0.76 versus 6.86 ± 0.56; RNFL: 107.53 ± 12.9 versus 93.38 ± 9.07 versus 100.09 ± 7.2), indicating better neuronal preservation despite the exposure to IL-1β. Although our sample was too small to test other statistical associations, higher values of both PI and MSSS were present in Arg/Arg carriers among patients with undetectable IL-1β (PI: Pro/Pro 0.16 +/- 0.13, Arg/Pro 0.14 +/- 0.09, Arg/Arg 0.21 +/- 0.17; MSSS: Pro/Pro 1.73 +/- 1.62, Arg/Pro 1.73 +/- 1.68, Arg/Arg 2.61 +/- 2.33).

## Discussion

Neuroinflammation in MS induces demyelination by lymphocyte/macrophage infiltration, microglial/astrocytic activation and cytokine/chemokine production [35]. Neurodegeneration independently accompanies inflammation since the early stages of MS, and involves not only overtly demyelinated areas, but also normal appearing white and grey matter [36, 37]. Noteworthy, in addition to focal axon damage induced by demyelination, neuronal damage seems also an early and independent event, directly triggered by glutamate and cytokine exposure [14, 38–40].

Aim of this study was to assess the role of p53 in the synaptic and neurotoxic effects of IL-1β and of TNF-α in patients with MS. Both inflammatory cytokines enhance glutamate transmission [4, 7, 41], and excessive glutamatergic signaling is detrimental for neuronal survival, through a process termed excitotoxicity [42, 43]. Here we found that IL-1β-, but not TNF-α-mediated synaptic and neurotoxic effects were mediated by p53, since its pharmacological inhibition prevented sEPSC frequency alteration and cell swelling caused by IL-1β in vitro. Neuronal swelling directly reflects changes in ion omeostasis and disruption of neuronal membrane potential [44], suggesting that the neurotoxic effects of IL-1β is a primary event, even independently of myelin damage. We found that IL-1β significantly increased the expression of p21 protein, which represents a canonical downstream target of activated p53 [33], indicating that activation of p53 pathway occurs after IL-1β exposure in the mouse brain. Furthermore, we identified TRPV1 channels as critical molecular targets of the action of p53 at glutamatergic synapses. TRPV1 channels are non-selective cation channels initially recognized to be activated by capsaicin, the pungent component of hot peppers [45], high temperature or low pH [46]. Early studies have supported a main pro-inflammatory role of TRPV1 channels, because mice lacking these channels show a reduction of pro-inflammatory cytokine release, and a decrease of inflammation-driven tissue damage [47–49]. We have previously demonstrated that these channels are essential mediators of the cellular effects of IL-1β [7], but, to our knowledge, this is the first evidence for a role in the synaptic effects of p53.

We also provided evidence for the involvement of PKC/TRPV1 pathway in the IL-1β-p53 interaction at glutamatergic synapses. In the presence of the TRPV1 antagonist capsazepine or of the PKC inhibitor chelerythrine, in fact, the action of p53 activator nutlin-3 on IL-1β synaptic effects was lost. Nutlins were identified as the first potent and specific small molecule Mdm2 antagonists that inhibit the p53-Mdm2 interaction, thus selectively increasing p53 levels and inducing apoptosis [50]. Nutlin-3 has been shown to limit the cellular effects of other inflammatory cytokines, like TNF-α, in neoplastic cells [51], but no previous evidence exists about the modulation of the IL-1β effects in neurons. Of note, IL-1β concentration used for in vitro experiments was in the range of ng/ml, but we have previously demonstrated that IL-1β CSF levels, although at lower concentration, are associated to functional changes of cortical excitability among MS patients [7].

The IL-1β-p53 interaction was also addressed in the inflammatory neurodegenerative process of MS patients. We investigated the possible relationship between the polymorphism at codon 72 of TP53 gene and the risk of late progression of neurological disability in subjects with RRMS, who underwent lumbar puncture to measure IL-1β levels. Our results indicate that this p53 polymorphism may affect disability progression in MS subjects by interacting with IL-1β signaling. In line with this, we found that the Pro/Pro genotype could abrogate the association between IL-1β and both clinical and morphological alterations, measured by disability scores and OCT parameters, respectively. Retinal alterations in MS patients accurately model the mechanisms of neurodegeneration in MS, and MV and RNFL thickness, obtained by OCT scans, are reliable measures of the integrity of, respectively, neurons and their axonal projections within the retina [52, 53]. In fact, a close relationship has been found between RNFL thickness and brain atrophy evaluated at the MRI in MS subjects [54].

The mechanism underlying the role of Arg72Pro polymorphism could be associated with the differential efficiency of transcription of p53-regulated genes in the three genotypes, as p53 with arginine at codon 72 favors apoptosis, whereas the Pro encoding allele exhibits a lower apoptotic potential [31]. In recent years, apoptotic biochemical cascades have been recognized to exert local actions on the functions and structural dynamics of growth cones and synapses, to play roles in neuronal plasticity [55, 56]. In particular, active caspases can be detected in cells that are not destined to die, and it is now widely accepted that caspases can play non-apoptotic roles in various pathological and physiological contexts [57–60]. Studies from several groups collectively point to an essential function of caspases in synaptic plasticity, independent of neuronal cell death. Accordingly, two initiator caspases (caspase-1 and caspase-9) and the effector caspase-3 are shown to regulate long-lasting synaptic plasticity in hippocampal neurons [57, 58]. Furthermore, an overexpression of p73, a protein that shares structural and functional homology with p53, is sufficient to induce neurite outgrowth [59], but more direct roles for p53 family members in regulating neurite outgrowth and synaptic modification have not been established before.

Emerging evidence indicates that apoptotic pathways are active in early stages of classical neurodegenerative diseases, such as Alzheimer disease [60] and inflammatory neurodegenerative diseases as MS (present work). The apoptotic cascade could mediate synapse dysfunction and loss, before the advent of cell death and neurodegeneration. These new insights have potential implications for neuroprotection, and highlight the regulatory role of p53 in the excitatory synaptic transmission under both normal and pathological conditions.

When immune challenge becomes chronic and/or unregulated, as in MS, the CNS can be chronically exposed to cytokines with resultant neuronal and axonal dysfunctions, later irreversible cellular damage and sustained neurological disability. Consistently, persistent presence of IL-1β has been associated with disability progression in RRMS [16].

Inflammatory cytokines can lead to neuronal death by driving excitotoxicity and/or activating apoptotic pathways, as our present data seem to indicate. However, IL-1β could not be the only significant factor to explain disability or poor prognosis in RRMS.

A limitation of this study was the small sample size. Further studies on larger samples of patients are warranted to replicate these data and to assess the effect of p53 per se on disability progression in MS. P53, in fact, could affect different pathways of apoptosis induction, thus leading to neurodegenerative damage in MS. In line with this, higher progression indexes were evident among carriers of the allele with higher apoptotic potential, regardless the presence of IL-1β. Nonetheless, our study provides compelling evidence of the synaptic mechanism linking cytokine signaling and excitotoxic neurodegeneration in MS, and suggest that targeting IL-1β-p53 effects on synaptic transmission might be neuroprotective in this severe disease.

## Methods

This study complied with the principles of the Declaration of Helsinki, and was approved by the Ethical Committee of the Policlinico Università Tor Vergata in Rome. All the subjects gave their written informed consent to the study. In regard to animal experiments, all efforts were made to minimize animal suffering and to reduce the number of mice used, in accordance with the European Communities Council Directive of November 24, 1986 (86/609/EEC).

### In vitro neurophysiology and cell swelling experiments

Female C57BL/6 mice were used for all the experiments. Six- to eight-week-old mice were killed by cervical dislocation under halothane anesthesia, and corticostriatal coronal slices (200 μm) were prepared from fresh tissue blocks of the brain using a vibratome [4, 7] A single slice was transferred to a recording chamber and submerged in a continuously flowing artificial CSF (aCSF) (34°C, 2–3 ml/min) gassed with 95% O_2_–5% CO_2_. The composition of the control aCSF was (in mM): 126 NaCl, 2.5 KCl, 1.2 MgCl_2_, 1.2 NaH_2_PO_4_, 2.4 CaCl_2_, 11 glucose, 25 NaHCO_3_. Whole cell patch clamp recordings were made in voltage clamp mode [4, 7], at the holding potential of -80 mV to study spontaneous glutamate-mediated sEPSCs. Drugs were applied by dissolving them to the desired final concentration in the bathing aCSF. One to 6 cells per animal were recorded. For each type of experiment, at least 4 distinct animals were employed. Throughout the text, ‘n’ refers to the number of cells, unless otherwise specified. To measure cell swelling [4], a baseline image of the cells was obtained and stored. The slice was then exposed to the investigated cytokine for the entire duration of the experiment. Individual neurons were visualized in situ using a differential interference contrast (Nomarski) optical system. This employed a 40× water immersion objective combined with an infrared filter, a monochrome charge-coupled device camera (COHU 4912; Cohu Electronics, Poway, CA), and a PC-compatible system for analysis of images and contrast enhancement (Winvision 2000; Delta Sistemi, Rosolina, Italy). Digital images were stored for subsequent analysis. Cells were typically visualized from 10 to 150 μm below the surface of the slice. To quantify changes in response to inflammatory cytokines, in the presence or in the absence of CNQX and PFT, cross-sectional somatic area was measured (IAS, Delta Sistemi). Each measurement was made 2 or 3 on individual medium-sized striatal neurons, and the average value was recorded at each time point. Drugs were applied by dissolving them to the desired final concentration in the bathing ACSF. Drugs were: bicuculline (10 μM), PFT (30 μM), PMA (2 μM)(from Sigma-RBI, St. Louis, USA); chelerythrine (10 μM) (from Alomone Labs); capsaicin (10nM), capsazepine (10 μM), CNQX (10 μM) (from Tocris Cookson, Bristol, UK), IL-1β (30 ng/ml), IL-1 receptor antagonist (IL-1ra; 10 μg/ml) (from R&D Systems, Minneapolis, USA); nutlin-3 (10 μM) (from Calbiochem, San Diego, USA), TNF-α (600 nM) (from Peprotech, Rocky Hill, USA). The purity of all the drugs used for the experiments was at least ≥95%, as reported in the manufacturer’s catalogue.

### Western blot analysis

Mouse corticostriatal slices of C57BL/6 J females were frozen after 2 h of incubation with medium or IL-1β 30 ng/ml. Tissues were transferred in microcentrifuge tubes containing RIPA buffer [50 mMTris-HCl pH 8, 200 mMNaCl, 2 mM EDTA, 1% NP-40, 0.5% sodium deoxycholate, 0.05% SDS, 1 mM Na_3_VO_4_, 1 mg/ml leupetin, 1 mg/ml aprotinin, 5 mM NaF, 1 mM PMSF freshly added] and homogenized using a pestle mounted in a motorized chuck. Homogenized tissues were left on ice for 15 mins, cell lysates were centrifuged for 15 mins at 15000 g at 4°C, and the supernatants were collected and used for Western blot analyses. Cell extracts were diluted in Laemmli buffer and boiled for 5 mins at 95°C. Proteins were separated on 12% SDS-PAGE gels and transferred to nitrocellulose (NC) membranes (Whatman, Sigma-Aldrich, GE Healthcare Life Science) using a wet blotting apparatus (Amersham Biosciences). Membranes were saturated for 1 h at room temperature with 5% non-fat dry milk in phosphate-buffered saline (PBS), containing 0.1% Tween-20. Membranes were incubated with the following antibodies overnight at 4°C: rabbit polyclonal anti-rat p21 (Santa Cruz Biotechnology; 1:500 dilution in 3% non-fat dry milk in PBS, containing 0.1% Tween-20), mouse monoclonal anti-rat β-actin (Sigma-Aldrich; 1:5000 dilution in 5% non-fat dry milk in PBS, containing 0.1% Tween-20). Secondary anti-rabbit or anti-mouse IgGs conjugated to horseradish peroxidase (Cell signaling) were incubated with the membranes for 1 h at room temperature at a 1:2000 dilution in PBS containing 0.1% Tween 20. Immunostained bands were detected by chemiluminescent method (Pierce ECL Western Blotting Substrate, Thermo Scientific).

### MS subjects and CSF withdrawal

Demographical features and clinical characteristics of RRMS patients are shown in Table 3. The minimum and maximum last EDSS values were respectively 0 and 6.5. All patients had received immunomodulatory treatment during the course of their disease. First-line treatment was started for all patients at the time of the diagnosis. All patients matched published criteria for the diagnosis of RRMS [61]. All were in clinical and radiological remission phase of the disease and naïve to any disease modifying treatment at the time of CSF withdrawal. In particular, subjects who experienced a clinical relapse within 60 days or showed any gadolinium (Gd)-enhanced lesions at brain magnetic resonance imaging (MRI) were excluded, as reported [16]. Enrolled subjects reported no previous history of chronic inflammatory diseases, or signs of local or systemic infection at the time of CSF collection. Lumbar puncture and brain MRI were performed within 24 h. Blood sample collection, CSF withdrawal and clinical assessments were performed at the MS Center of the Tor Vergata University Hospital of Rome by MS specialist neurologists. Demographic and clinical information were derived from medical records. MS disease onset was defined as the first episode of focal neurological dysfunction indicative of MS. Disease duration was estimated as the number of years from onset to the last assessment of disability. At the time of confirmed diagnosis, all MS patients had started first-line disease-modifying therapies, without differences comparing between patients with undetectable and detectable CSF levels of IL-1β (IL-1β+: daily glatiramer acetate 20 mg s.c. n = 28/77, interferon beta 1a 44 mcg s.c. three times weekly n = 34/77, interferon beta 1a 30 mcg i.m. n = 5/77, or interferon beta 1b 250 mcg s.c. every other day n = 10/77; IL-1β-: glatiramer acetate n = 35/93, interferon beta 1a 44 mcg n = 39/93, interferon beta 1a 30 mcg n = 6/93, interferon beta 1b n = 13/93).

**Table 3:** **Demographic and clinical characteristics of MS subjects, according to CSF contents of IL-1β**

		IL-1β		
	Total	+	-	p
Number	170	77	93	
Gender (M/F)	62/108	27/50	35/58	0.75
Age (years)	36.3 ± 9.5	37.3 ± 10.1	35.1 ± 8.8	0.12
Disease duration (years)	10.5 ± 5.3	10.8 ± 5.9	10.1 ± 4.8	0.42
EDSS	2.2 ± 1.7	2.8 ± 1.9	1.7 ± 1.4	<0.01

+, detectable; -, undetectable; M, male; F, female; EDSS, Expanded Disability Status Scale.

### IL-1β determination

To determine IL-1β, the CSF was centrifuged and immediately stored at -80°C until analyzed using Bio-Plex Multiplex Cytokine Assay (Bio-Rad Laboratories, Hercules, CA), according to manufacturer’s instructions. Concentrations of IL-1β were calculated according to a standard curve and expressed as picograms per milliliter. When the concentrations of the cytokine were below the detection threshold, they were assumed to be 0 pg/ml [16].

### Disability assessment

Disability was determined by a specially trained (Neurostatus, 2006. Available at http://www.neurostatus.net) and certified examining neurologist using Expanded Disability Status Scale (EDSS), a 10-point disease severity score derived from nine ratings for individual neurological domains [62]. The EDSS, evaluated every six months since diagnosis, was used in combination with disease duration to calculate two measures of disease severity, the PI and the MSSS. PI was defined as EDSS/disease duration. The MSSS is an algorithm that relates EDSS scores to distribution of disability in patients with comparable disease durations [63]. EDSS scores were taken in account for the assessment of disability progression when obtained at least 30 days since stabilization/resolution of previous relapse and/or corticosteroid treatment. Only patients with at least four years of follow-up were included.

### Optical coherence tomography

Medical history with respect to visual symptoms was taken from all MS subjects. Self-report and physician report were confirmed by record review. A subset of RR MS patients (n = 118) without history of optic neuritis and ophthalmological disease underwent measurement of RNFL thickness and MV for both eyes using Stratus OCT™; software version 4.0.2, Carl Zeiss Meditec, Inc.) [64]. Briefly, for MV, retinal thickness was measured automatically as the distance between the vitreoretinal interface and the anterior boundary of the retinal pigment epithelium. Stratus OCT images were generated using the fast map scan protocol consisting of six radial scans spaced 30° apart, with each scan measuring 6 mm in length. Each image had a resolution of 10 μm axially and 20 μm transversally. All Stratus OCT images had a signal strength of 6 μm. RNFL thickness measurements were read from the automated measurements generated by the machine using the Fast RNFL analysis. For the study scanning was performed after pharmacological dilation. Average RNFL thickness for 360° around the optic disc was recorded. Values were adjusted for age. One randomly chosen eye from each subject was included in the study. Testing was performed by trained technicians experienced in examination of patients for research studies, and patients wore their habitual glasses or contact lenses for distance correction.

### Genotyping

Genomic DNA was extracted from peripheral blood lymphocytes of patients. Genotyping was performed using MassARRAY high-throughput DNA analysis with Matrix-assisted laser desorption/ionization time-of-flight (MALDI-TOF) mass spectrometry (Sequenom, Inc., San Diego, CA). SNP (G-to-C transversion) at codon 72 (rs1042522) of the gene encoding p53 (TP53) was genotyped using iPLEX Gold technology following manufacturer protocol (Sequenom). Detailed description of methods has been previously reported [65].

### Statistical analysis

For electrophysiological data, statistical analysis was performed using a paired or unpaired Student’s t-test if comparisons were between two groups. Multiple comparisons were analyzed by one-way ANOVA for independent and/or repeated measures followed by Tukey HSD. MS subjects were divided into two groups according to the detectable (+ group) or undetectable (- group) CSF levels of IL-1β. Differences among groups were compared by univariate analysis using Student’s t test or Mann–Whitney test for continuous variables and Fisher Exact Test or Chi-square Test for categorical variables.

Patient characteristics according to CSF IL-1β contents were shown in Table 3. The 3 (genotypes) × 2 (cytokine detection) between subjects analysis of covariance, was performed to investigate the interaction effects between CSF IL-1β and TP53 genotype, with disease duration as covariate for ophthalmologic parameters. The 2 (genetic model) × 2 (cytokine detection) between subjects analyses of covariance, with disease duration as covariate, was performed to evaluate the effects of CSF IL-1β on dominant (Arg/Arg vs. Arg/Pro + Pro/Pro) and recessive (Arg/ Arg + Arg/Pro vs. Pro/Pro) models. The 3 × 2 and 2 × 2 ANOVAs were replicated for disability progression indexes.

Electrophysiological data were presented as the mean ± standard error (SE). Other data were presented as mean ± standard deviation (SD). A p-value (p) of less than 0.05 was considered statistically significant. The threshold for significance applied throughout of the present study was not taking into account the number of comparisons performed.

## Abbreviations

aCSF: Artificial CSF
CNQX: Cyano-nitroquinoxaline-dione
CSF: Cerebrospinal fluid
EDSS: Expanded disability status scale
IL-1β: Interleukin-1β
IL-1ra: IL-1 receptor antagonist
MALDI-TOF: Matrix-assisted laser desorption/ionization time-of-flight
MRI: Magnetic resonance imaging
MS: Multiple sclerosis
MSSS: MS Severity Scale
MV: Macular volume
NC: Nitrocellulose
OCT: Optical coherence tomography
PBS: Phosphate-buffered saline
PFT: Pifithrin-α
PI: Progression Index
PKC: Protein kinase C
PMA: Phorbol 12-myristate 13-acetate
RNFL: Retinal nerve fibre layer
RRMS: Relapsing-remitting MS
SD: Standard deviation
SE: Standard error
sEPSC: Spontaneous excitatory post-synaptic current
SNP: Single nucleotide polymorphism
TNF-α: Tumor necrosis factor
TRPV1: Transient receptor potential vanilloid 1.

## References

[1] Cosentino M, Fietta AM, Ferrari M, Rasini E, Bombelli R, Carcano E, Saporiti F, Meloni F, Marino F, Lecchini S (2007). Human CD4 + CD25+ regulatory T cells selectively express tyrosine hydroxylase and contain endogenous catecholamines subserving an autocrine/paracrine inhibitory functional loop. Blood.

[2] Fazzino F, Urbina M, Cedeño N, Lima L (2009). Fluoxetine treatment to rats modifies serotonin transporter and cAMP in lymphocytes, CD4+ and CD8+ subpopulations and interleukins 2 and 4. Int Immunopharmacol.

[3] Saha B, Mondal AC, Majumder J, Basu S, Dasgupta PS (2001). Physiological concentrations of dopamine inhibit the proliferation and cytotoxicity of human CD4+ and CD8+ T cells in vitro: a receptor-mediated mechanism. Neuroimmunomodulation.

[4] Centonze D, Muzio L, Rossi S, Cavasinni F, De Chiara V, Bergami A, Musella A, D’Amelio M, Cavallucci V, Martorana A, Bergamaschi A, Cencioni MT, Diamantini A, Butti E, Comi G, Bernardi G, Cecconi F, Battistini L, Furlan R, Martino G (2009). Inflammation triggers synaptic alteration and degeneration in experimental autoimmune encephalomyelitis. J Neurosci.

[5] Haji N, Mandolesi G, Gentile A, Sacchetti L, Fresegna D, Rossi S, Musella A, Sepman H, Motta C, Studer V, De Chiara V, Bernardi G, Strata P, Centonze D (2012). TNF-α-mediated anxiety in a mouse model of multiple sclerosis. Exp Neurol.

[6] Raison CL, Borisov AS, Woolwine BJ, Massung B, Vogt G, Miller AH (2010). Interferon-alpha effects on diurnal hypothalamic-pituitary-adrenal axis activity: relationship with proinflammatory cytokines and behavior. Mol Psychiatry.

[7] Rossi S, Furlan R, De Chiara V, Motta C, Studer V, Mori F, Musella A, Bergami A, Muzio L, Bernardi G, Battistini L, Martino G, Centonze D (2012). Interleukin-1β causes synaptic hyperexcitability in multiple sclerosis. Ann Neurol.

[8] Rossi S, Sacchetti L, Napolitano F, De Chiara V, Motta C, Studer V, Musella A, Barbieri F, Bari M, Bernardi G, Maccarrone M, Usiello A, Centonze D (2012). Interleukin-1β causes anxiety by interacting with the endocannabinoid system. J Neurosci.

[9] Rossi S, Studer V, Motta C, De Chiara V, Barbieri F, Bernardi G, Centonze D (2012). Inflammation inhibits GABA transmission in multiple sclerosis. Mult Scler.

[10] Zhu CB, Blakely RD, Hewlett WA (2006). The proinflammatory cytokines interleukin-1beta and tumor necrosis factor-alpha activate serotonin transporters. Neuropsychopharmacology.

[11] Ellwardt E, Zipp F (2014). Molecular mechanisms linking neuroinflammation and neurodegeneration in MS. Exp Neurol.

[12] Millington C, Sonego S, Karunaweera N, Rangel A, Aldrich-Wright R, Campbell IL, Gyengesi E, Münch G (2014). Chronic neuroinflammation in Alzheimer’s disease: new perspectives on animal models and promising candidate drugs. Biomed Res Int.

[13] Froger N, Orellana JA, Calvo CF, Amigou E, Kozoriz MG, Naus CC, Sáez JC, Giaume C (2010). Inhibition of cytokine-induced connexin43 hemichannel activity in astrocytes is neuro- protective. Mol Cell Neurosci.

[14] Lai AY, Swayze RD, El-Husseini A, Song C (2006). Interleukin-1 beta modulates AMPA receptor expression and phosphorylation in hippocampal neurons. J Neuroimmunol.

[15] Tolosa L, Caraballo-Miralles V, Olmos G, Lladó J (2011). TNF-α potentiates glutamate-induced spinal cord motoneuron death via NF-kB. Mol Cell Neurosci.

[16] Rossi S, Studer V, Motta C, Germani G, Macchiarulo G, Buttari F, Mancino R, Castelli M, De Chiara V, Weiss S, Martino G, Furlan R, Centonze D (2014). Cerebrospinal fluid detection of interleukin-1β in phase of remission predicts disease progression in multiple sclerosis. J Neuroinflammation.

[17] Espín R, Roca FJ, Candel S, Sepulcre MP, González-Rosa JM, Alcaraz-Pérez F, Meseguer J, Cayuela ML, Mercader N, Mulero V (2013). TNF-α receptors regulate vascular homeostasis in zebra fish through a caspase-8, caspase-2 and P53 apoptotic program that by passes caspase-3. Dis Model Mech.

[18] Goretsky T, Dirisina R, Sinh P, Mittal N, Managlia E, Williams DB, Posca D, Ryu H, Katzman RB, Barrett TA (2012). p53 mediates TNF-α-induced epithelial cell apoptosis in IBD. Am J Pathol.

[19] Nalca A, Rangnekar VM (1998). The G1-phase growth-arresting action of interleukin-1 is independent of p53 and p21/WAF1 function. J Biol Chem.

[20] Song K, Fukushima P, Seth P, Sinha BK (1998). Role of p53 and apoptosis in sensitization of cis-diammine dichloroplatinum antitumor activity by interleukin-1 in ovarian carcinoma cells. Int J Oncol.

[21] Vadrot N, Ghanem S, Braut F, Gavrilescu L, Pilard N, Mansouri A, Moreau R, Reyl-Desmars F (2012). Mitochondrial DNA maintenance is regulated in human hepatoma cells by glycogen synthase kinase 3β and p53 in response to tumor necrosis factor α. PLoS One.

[22] Wang C, Wang MW, Tashiro S, Onodera S, Ikejima T (2005). Evodiamine induced human melanoma A375-S2 cell death partially through interleukin 1 mediated pathway. Biol Pharm Bull.

[23] Aloyz RS, Bamji SX, Pozniak CD, Atwal J, Kaplan DR (1998). p53 is essential for developmental neuron death as regulated by TrkA and p75 neurotrophin receptors. J Cell Biol.

[24] Alves da Costa C, Paitel E, Mattson MP, Amson R, Telerman A, Ancolio K, Checler F (2002). Wild-type and mutated presenilins 2 trigger p53-dependent apop- tosis and down regulate presenilin 1 expression in HEK293 human cells and in murine neurons. Proc Natl Acad Sci U S A.

[25] Culmsee C, Zhu X, Yu QS, Chan SL, Camandola S, Guo Z, Greig NH, Mattson MP (2001). A synthetic inhibitor of p53 protects neurons against death induced by ischemic and excitotoxic insults, and amyloid beta-peptide. J Neurochem.

[26] Hong LZ, Zhao XY, Zhang HL (2010). p53-mediated neuronal cell death in ischemic brain injury. Neurosci Bull.

[27] Morrison RS, Kinoshita Y (2000). The role of p53 in neuronal cell death. Cell Death Differ.

[28] Bergamaschi D, Samuels Y, Sullivan A, Zvelebil M, Breyssens H, Bisso A, Del Sal G, Syed N, Smith P, Gasco M, Crook T, Lu X (2006). iASPP preferentially binds p53 proline-rich region and modulates apoptotic function of codon 72-polymorphic p53. Nat Genet.

[29] Bonafé M, Salvioli S, Barbi C, Trapassi C, Tocco F, Storci G, Invidia L, Vannini I, Rossi M, Marzi E, Mishto M, Capri M, Olivieri F, Antonicelli R, Memo M, Uberti D, Nacmias B, Sorbi S, Monti D, Franceschi C (2004). The different apoptotic potential of the p53 codon 72 alleles increases with age and modulates in vivo ischaemia-induced cell death. Cell Death Differ.

[30] Dumont P, Leu JI, Della Pietra AC, George DL, Murphy M (2003). The codon 72 polymorphic variants of p53 have markedly different apoptotic potential. Nat Genet.

[31] Jeong BS, Hu W, Belyi V, Rabadan R, Levine AJ (2010). Differential levels of transcription of p53-regulated genes by the arginine/proline polymorphism: p53 with arginine at codon 72 favors apoptosis. FASEB J.

[32] Musella A, De Chiara V, Rossi S, Prosperetti C, Bernardi G, Maccarrone M, Centonze D (2009). TRPV1 channels facilitate glutamate transmission in the striatum. Mol Cell Neurosci.

[33] Weinberg WC, Denning MF (2002). P21Waf1 control of epithelial cell cycle and cell fate. Crit Rev Oral Biol Med.

[34] Hock AK, Vigneron AM, Carter S, Ludwig RL, Vousden KH (2011). Regulation of p53 stability and function by the deubiquitinating enzyme USP42. EMBO J.

[35] Sospedra M, Martin R (2005). Immunology of multiple sclerosis. Annu Rev Immunol.

[36] Geurts JJ, Barkhof F (2008). Grey matter pathology in multiple sclerosis. Lancet Neurol.

[37] Zeis T, Graumann U, Reynolds R, Schaeren-Wiemers N (2008). Normal-appearing white matter in multiple sclerosis is in a subtle balance between inflammation and neuroprotection. Brain.

[38] Centonze D, Muzio L, Rossi S, Furlan R, Bernardi G, Martino G (2010). The link between inflammation, synaptic transmission and neurodegeneration in multiple sclerosis. Cell Death Differ.

[39] Choi DW (1988). Glutamate neurotoxicity and diseases of the nervous system. Neuron.

[40] Stellwagen D, Malenka RC (2006). Synaptic scaling mediated by glial TNF-alpha. Nature.

[41] Mandolesi G, Musella A, Gentile A, Grasselli G, Haji N, Sepman H, Fresegna D, Bullitta S, De Vito F, Musumeci G, Di Sanza C, Strata P, Centonze D (2013). Interleukin-1β alters glutamate transmission at purkinje cell synapses in a mouse model of multiple sclerosis. J Neurosci.

[42] Forder JP, Tymianski M (2009). Postsynaptic mechanisms of excitotoxicity: Involvement of postsynaptic density proteins, radicals, and oxidant molecules. Neuroscience.

[43] Olney JW (1969). Brain lesions, obesity, and other disturbances in mice treated with monosodium glutamate. Science.

[44] Centonze D, Prosperetti C, Barone I, Rossi S, Picconi B, Tscherter A, De Chiara V, Bernardi G, Calabresi P (2006). NR2B-containing NMDA receptors promote the neurotoxic effects of 3-ritropropionic acid but not of rotenone in the striatum. Exp Neurol.

[45] Tominaga M, Caterina MJ, Malmberg AB, Rosen TA, Gilbert H, Skinner K, Raumann BE, Basbaum AI, Julius D (1998). The capsaicin receptor integrates multiple pain producing stimuli. Neuron.

[46] Caterina MJ, Schumacher MA, Tominaga M, Rosen TA, Levine JD, Julius D (1997). The capsaicin receptor: a heat-activated ion channel in the pain pathway. Nature.

[47] Caterina MJ, Leffler A, Malmberg AB, Martin WJ, Trafton J, Petersen-Zeitz KR (2000). Impaired nociception and pain sensation in mice lacking the capsaicin receptor. Science.

[48] Keeble J, Russell F, Curtis B, Starr A, Pintér E, Brain SD (2005). Involvement of transient receptor potential vanilloid 1 in the vascular and hyperalgesic components of joint inflammation. Arthritis Rheum.

[49] Tóth DM, Szőke E, Bölcskei K, Kvell K, Bender B, Bősze Z, Szolcsányi J, Sándor Z (2011). Nociception, neurogenic inflammation and thermoregulation in TRPV1 knockdown transgenic mice. Cell Mol Life Sci.

[50] Kojima K, Konopleva M, McQueen T, O’Brien S, Plunkett W, Andreeff M (2006). Mdm2 inhibitor Nutlin-3a induces p53-mediated apoptosis by transcription-dependent and transcription-independent mechanisms and may overcome Atm mediated resistance to fludarabine in chronic lymphocytic leukemia. Blood.

[51] Dey A, Wong ET, Bist P, Tergaonkar V, Lane DP (2007). Nutlin-3 inhibits the NFkappaB pathway in a p53-dependent manner: implications in lung cancer therapy. Cell Cycle.

[52] Frohman EM, Fujimoto JG, Frohman TC, Calabresi PA, Cutter G, Balcer LJ (2008). Optical coherence tomography: a window into the mechanisms of multiple sclerosis. Nat Clin Pract Neurol.

[53] Barkhof F, Calabresi PA, Miller DH, Reingold SC (2009). Imaging outcomes for neuroprotection and repair in multiple sclerosis trials. Nat Rev Neurol.

[54] Burkholder BM, Osborne B, Loguidice MJ, Bisker E, Frohman TC, Conger A, Ratchford JN, Warner C, Markowitz CE, Jacobs DA, Galetta SL, Cutter GR, Maguire MG, Calabresi PA, Balcer LJ, Frohman EM (2009). Macular volume determined by optical coherence tomography as a measure of neuronal loss in multiple sclerosis. Arch Neurol.

[55] Gilman CP, Mattson MP (2002). Do apoptotic mechanisms regulate synaptic plasticity and growth-cone motility?. Neuromol Med.

[56] Li Z, Sheng M (2012). Caspases in synaptic plasticity. Mol Brain.

[57] Li Z, Jo J, Jia JM, Lo SC, Whitcomb DJ, Jiao S, Cho K, Sheng M (2010). Caspase-3 activation via mitochondria is required for long-term depression and AMPA receptor internalization. Cell.

[58] Lu C, Wang Y, Furukawa K, Fu W, Ouyang X, Mattson MP (2006). Evidence that caspase-1 is a negative regulator of AMPA receptor-mediated long-term potentiation at hippocampal synapses. J Neurochem.

[59] De Laurenzi V, Raschella G, Barcaroli D, Annicchiarico-Petruzzelli M, Ranalli M, Catani MV, Tanno B, Costanzo A, Levrero M, Melino G (2000). Induction of neuronal differentiation by p73 in a neuroblastoma cell line. J Biol Chem.

[60] D’Amelio M, Cavallucci V, Middei S, Marchetti C, Pacioni S, Ferri A, Diamantini A, De Zio D, Carrara P, Battistini L, Moreno S, Bacci A, Ammassari-Teule M, Marie H, Cecconi F (2011). Caspase-3 triggers early synaptic dysfunction in a mouse model of Alzheimer’s disease. Nat Neurosci.

[61] Polman CH, Reingold SC, Edan G, Filippi M, Hartung HP, Kappos L, Lublin FD, Metz LM, McFarland HF, O’Connor PW, Sandberg-Wollheim M, Thompson AJ, Weinshenker BG, Wolinsky JS (2005). Diagnostic criteria for multiple sclerosis: 2005 revisions to the “McDonald Criteria”. Ann Neurol.

[62] Kurtzke JF (1983). Rating neurologic impairment in multiple sclerosis: an expanded disability status scale (EDSS). Neurology.

[63] Roxburgh RH, Seaman SR, Masterman T, Hensiek AE, Sawcer SJ, Vukusic S, Achiti I, Confavreux C, Coustans M, le Page E, Edan G, McDonnell GV, Hawkins S, Trojano M, Liguori M, Cocco E, Marrosu MG, Tesser F, Leone MA, Weber A, Zipp F, Miterski B, Epplen JT, Oturai A, Sørensen PS, Celius EG, Lara NT, Montalban X, Villoslada P, Silva AM (2005). Multiple Sclerosis Severity Score: using disability and disease duration to rate disease severity. Neurology.

[64] Rossi S, Mancino R, Bergami A, Mori F, Castelli M, De Chiara V, Studer V, Mataluni G, Sancesario G, Parisi V, Kusayanagi H, Bernardi G, Nucci C, Bernardini S, Martino G, Furlan R, Centonze D (2011). Potential role of IL-13 in neuroprotection and cortical excitability regulation in multiple sclerosis. Mult Scler.

[65] Hasan SK, Buttari F, Ottone T, Voso MT, Hohaus S, Marasco E, Mantovani V, Garagnani P, Sanz MA, Cicconi L, Bernardi G, Centonze D, Lo-Coco F (2011). Risk of acute promyelocytic leukemia in multiple sclerosis: coding variants of DNA repair genes. Neurology.

